# A multicenter prospective audit to investigate the current management of patients undergoing anti-reflux surgery in the UK: Audit & Review of Anti-Reflux Operations & Workup

**DOI:** 10.1093/dote/doaa129

**Published:** 2021-01-16

**Authors:** The ARROW Study Group, Rob Walker, Tom Wiggins, Natalie S Blencowe, John M Findlay, Michael Wilson, Andrew C Currie, Steve Hornby, Sheraz R Markar, Saqib Rahman, Megan Lloyd, Marianne Hollyman, Shameen Jaunoo

**Affiliations:** Cancer Sciences, University of Southampton, Southampton, UK; Cancer Sciences, University of Southampton, Southampton, UK; Cancer Sciences, University of Southampton, Southampton, UK; Cancer Sciences, University of Southampton, Southampton, UK; Cancer Sciences, University of Southampton, Southampton, UK; Cancer Sciences, University of Southampton, Southampton, UK; Cancer Sciences, University of Southampton, Southampton, UK; Cancer Sciences, University of Southampton, Southampton, UK; Cancer Sciences, University of Southampton, Southampton, UK; Cancer Sciences, University of Southampton, Southampton, UK; Cancer Sciences, University of Southampton, Southampton, UK; Cancer Sciences, University of Southampton, Southampton, UK

**Keywords:** anti-reflux surgery, clinical audit (MeSH), fundoplication (MeSH), gastroesophageal reflux (MeSH), hiatal hernia (MeSH)

## Abstract

**Background:**

There are a variety of surgical and endoscopic interventions available to treat gastroesophageal reflux disease. There is, however, no consensus on which approach is best.

The aim of this national audit is to describe the current variation in the UK clinical practice in relation to anti-reflux surgery (ARS) and to report adherence to available clinical guidelines.

**Methods:**

This national audit will be conducted at centers across the UK using the secure online web platform ALEA. The study will comprise two parts: a registration questionnaire and a prospective multicenter audit of ARS. All participating centers will be required to complete the registration questionnaire comprising details regarding pre-, peri-, and post-operative care pathways and whether or not these are standardized within each center. Following this, a 12-month multicenter prospective audit will be undertaken to capture data including patient demographics, predominant symptoms, preoperative investigations, surgery indication, intraoperative details, and postoperative outcomes within the first 90 days.

Local teams will retain access to their own data to facilitate local quality improvement. The full dataset will be reported at national and international scientific congresses and will contribute to peer-reviewed publications and national quality improvement initiatives.

**Conclusions:**

This study will identify and explore variation in the processes and outcomes following ARS within the UK using a collaborative cohort methodology. The results generated by this audit will facilitate local and national quality improvement initiatives and generate new possibilities for future research in anti-reflux interventions.

## INTRODUCTION

Gastroesophageal reflux disease (GERD) is a common condition, affecting 10–20% of the Western population.^1^^,^^2^ In addition to having a detrimental effect on the quality of life, GERD is a risk factor for the development of Barrett’s esophagus^3^^,^^4^ and esophageal adenocarcinoma.^5^ Primary treatments include lifestyle modification and proton pump inhibitors (PPIs) which are generally well tolerated. Some patients continue to have refractory symptoms and others cannot tolerate, or do not wish to take, long-term medication. In these cases, anti-reflux surgery (ARS) may be a therapeutic option.^6–8^ Current guidelines from the National Institute of Health and Care Excellence (NICE) reflect this, with consideration of laparoscopic fundoplication recommended for patients with a confirmed diagnosis of acid reflux and who are not suitable for long-term acid suppression therapy.^9^

Despite national guidelines and published evidence from randomized controlled trials,^10–12^ there is a lack of consensus regarding the most effective ARS technique, and whether procedures should be tailored to a particular patient’s symptomology, nature of reflux disease, or esophageal motility. Technical uncertainties in fundoplication (the most common procedure) include the extent of dissection (i.e. hiatal dissection and division of short gastric vessels),^11^ wrap formation (i.e. partial, full, anterior or posterior),^12–14^ whether gastropexy is required,^15^^,^^16^ and the method of crural repair^17^ (including whether this should be undertaken at all, and whether mesh should be utilized to reinforce the repair).^18^ In addition to fundoplication, other minimally invasive techniques such as LINX™,^19^ Stretta™,^20^ and EsophyX™^21^ are available, although the precise role of these novel treatments is currently unclear (Supplementary A1). There is also anecdotal inconsistency in the selection of patients for surgery and in the use of preoperative assessment investigations, despite recommendations from the Association of Upper GI Surgeons (AUGIS),^22^ British Society of Gastroenterology (BSG),^23^ and the recent International Consensus Regarding Preoperative Examinations and Clinical Characteristics Assessment to Select Adult Patients for Antireflux Surgery (ICARUS) guidelines.^24^

Strong recommendations from the ICARUS and BSG guidelines include the need for esophageal manometry to be performed prior to consideration of ARS.^23^^,^^24^ The primary purpose of esophageal manometry is to identify any major esophageal motility disorder, gastro-esophageal outflow obstruction, or absence of contractility, in order to prevent ARS from being performed in patients with a primary motility disorder such as achalasia or diffuse esophageal spasm.^23^^,^^24^ Other recommendations include the need for preoperative esophagogastroduodenoscopy within 12-months of ARS in order to identify the presence of Barrett’s esophagus (and grade dysplasia where present) and assess the size and configuration of any hiatal hernia.^24^

A previous study has highlighted significant variation in the UK in relation to the provision of ARS, although clinical outcomes were comparable.^25^ Variations included the rate of conversion to open procedures, 30-day reintervention or readmission, and rates of other adverse events. Unplanned readmission or reoperation have been identified as useful quality measures, as they may represent problems relating to the primary procedure itself.^26^^,^^27^ AUGIS have provided specific recommendations that all units performing ARS should have a rate of conversion to open surgery of under 5%, 30-day readmission rate of under 10%, and rate of unplanned return to theatre within 30-days of less than 5%.^22^

The aim of this national audit is to describe the current variation in UK clinical practice in relation to ARS, to compare adherence to current guidelines, and report short-term outcome measures (readmission and reoperation rate). The study will focus on patient selection, preoperative investigations, operative procedure and techniques, postoperative care, and short-term outcomes. This variation will be compared to recommendations from national and international guidelines.^22–24^

## METHODS AND ANALYSIS

This UK wide multicenter study will assess variation in the practice of ARS and compare this practice against a number of reported quality standards (Table 1).

**Table 1:** Details of audit standards utilized for the purposes of the current study

Source	Measure	Evidence	Expectation
British Society of Gastroenterology (BSG) Guidelines^23^ ICARUS Guidelines^24^	Esophageal manometry is mandatory in the work-up of patients for anti-reflux surgery	Documentation in patient care record	100%
ICARUS Guidelines^24^	In patients with non-erosive GERD Reflux monitoring is mandatory in the work-up of patients for anti-reflux surgery	Documentation in patient care record	100%
ICARUS Guidelines^24^	Endoscopy is mandatory in the work-up of patients for anti-reflux surgery and has to be carried out in the last year prior to anti-reflux surgery	Documentation in patient care record	100%
The Provision Of Services For Upper Gastrointestinal Surgery AUGIS^22^	Patients undergoing anti-reflux surgery should have this procedure completed laparoscopically (Unit level:<5% open conversion rate)	Documentation in patient care record	95%
The Provision Of Services For Upper Gastrointestinal Surgery AUGIS^22^	Patients undergoing anti-reflux surgery should not have an unplanned readmission (unit level <10% readmission rate at 30 days postoperatively)	Documentation in patient care record	90% (unit level)
The Provision Of Services For Upper Gastrointestinal Surgery AUGIS^22^	Patients undergoing anti-reflux surgery should not have an unplanned reoperation (unit level <5% reoperation rate at 30 days postoperatively)	Documentation in patient care record	95%

Although other recommendations are available within each of these clinical guidelines, these audit standards were strongly endorsed by the reporting guidelines as listed above.^22–24^ Other recommendations within these guidelines were not selected for measurement within the present audit as they may have related to patient selection (and therefore not possible to capture data from those patients not selected for ARS based on the current methodology), or were considered subjective and therefore difficult to define as a specific audit standard. Full details of all standards reported within these guidelines are provided in Supplementary A2, alongside details of the rationale for excluding those which were not included as specific audit standards for the current study.

### Study group

The study has been devised following a research development meeting held under the oversight of Royal College of Surgeons and AUGIS into unmet research need in upper gastrointestinal (UGI) Surgery. The study group has been formed of UGI surgeons and trainees who have expressed interest in this project and are operating under the umbrella of AUGIS and the Roux Group (AUGIS trainee body).

### Study approach

The study will comprise two parts: a registration questionnaire and a prospective multicenter audit of laparoscopic ARS.

All participating centers will be required to complete the registration questionnaire (Supplementary A3), comprising specific details about pre-, peri-, and post-operative care pathways and whether or not these are standardized within each center. Following this, a 12-month multicenter prospective audit will be undertaken.

#### Local registration with institution audit department

Each center will be responsible for registering the ARROW study with their local audit department. Research ethics approval is not required for this study, as confirmed by the NHS Health Research Authority decision tool (http://www.hra-decisiontools.org.uk/research/, accessed 13 December 2019, Supplementary A4). Inclusion in this study will not have any effect on an individual patient’s clinical pathway.

### Eligible patients

All patients aged 18 and over, undergoing primary or revisional ARS (and/or paraesophageal hernia repair for reflux symptoms) of any type will be eligible for inclusion. As well as fundoplication, patients undergoing LINX™, Stretta™, or EsophyX™ for reflux symptoms will be eligible for inclusion. Patients undergoing gastric bypass surgery following a primary referral for management of reflux symptoms will also be eligible for inclusion. Those referred initially as part of a weight-management pathway will be excluded, as will individuals undergoing conversion to gastric bypass for reflux following previous bariatric surgery. Patients undergoing ARS as part of the treatment of a non-reflux-related upper gastrointestinal condition (such as during treatment for achalasia or upper gastrointestinal cancer) will be excluded. Patients undergoing paraesophageal hernia repair for non-reflux related symptoms will also be excluded.

Patients will be identified from theatre scheduling systems, multidisciplinary team meetings, and co-ordination with the lead upper gastrointestinal (UGI) surgeon in each center.

### Eligible centers and surgeons

All centers and surgeons undertaking ARS will be eligible to take part. Currently, 83 centers have been recruited to participate. There will be no restrictions on the volume of practice. Centers within the National Health Service and private healthcare sector will be eligible for inclusion. Eligible centers will be identified through the National Research Collaborative network, individual surgical trainee research collaboratives (which encompass hospitals from most areas of the UK), AUGIS including the Roux Group (AUGIS trainee association), and the British Society of Gastroenterology. In regions without collaboratives, specific trainees will be targeted in order to ensure coverage from all areas of the UK.

Previous studies estimated that 2400 anti-reflux procedures are completed in England per year.^25^ We aim to capture a minimum of 25% of procedures over a 12-month period (with the additional benefit of collecting data from centers in the rest of the UK not included in previous Hospital Episode Statistics databases). We anticipate a minimum of 600 procedures to be recorded in this dataset. The number of cases recorded will not be capped and as additional centers not reflected in the Hospital Episode Statistics database figures will be recruited we hope that this minimum number of 600 procedures will be exceeded.

Each center will have a nominated lead surgeon who will be assisted by other team members to undertake patient identification, collection of a full dataset, and entry into the study registry. Prior to publication, each lead surgeon will be responsible for collating a list of contributors from their site.

### Data collection

Details of the data collection form are provided in Supplementary A5. Data will be collected in the following categories:

- Demographic details.
- Referral details.
- Predominant symptoms.
- Preoperative investigations.
- Indications for surgery.
- Intraoperative details.
- Postoperative details.
- Details of any readmission.
- Postoperative patient outcomes at the last follow-up point.

Clinical information regarding primary indication for surgery and nature of symptom profile identify whether patients experience typical or atypical symptoms of GERD and the relative response to PPIs. This will also allow patients to be classified according to criteria established by Sifrim *et al.*^28^ Clinical data will also include patient body mass index. Preoperative endoscopy results will also be collected with any hiatal hernia classified according to the measurements recorded at time of endoscopy as small (<2 cm) medium (2–5 cm), or large (>5 cm) and cross-references against size recorded in any imaging studies (contrast swallow or cross-sectional imaging). If present Barrett’s Esophagus will be classified according to the Prague criteria where recorded.^29^^,^^30^

### Data management

ARROW will use ALEA Clinical (www.aleaclinical.eu) to host electronic records. Collaborators will be granted online access to the survey section of the study and on completion of the survey and evidence that they have registered ARROW with their local audit department, they will be provided access to the prospective patient entry section. Collaborators will be asked to complete electronic Case Report Forms (eCRFs) for each patient in a timely manner using source documents from each individual case. In the event that records have not been updated for a period of 30-days a reminder e-mail will be sent to collaborators in order to ensure these are completed.

Data will be collected and retained in accordance with local laws and regulations, for example, the General Data Protection Regulation (2018) in the UK. The Lead Surgeon at each site is responsible for ensuring the accuracy, completeness, and timeliness of the data. Any study documents will be retained in a secure central location at the University of Southampton during and after the study has finished. During the study, all data will be reported in pseudo-anonymized form and identified by the assigned participant number. Individual sites will only have access on ALEA to data collected via their specific site, including that which links a participant to their assigned participant number. The administrative center (University of Southampton) will have access via ALEA to all data except that which links a participant to their assigned participant number, from all investigation sites. Ultimate responsibility for security and safety of data submitted to ALEA rests with Professor Tim Underwood and the University of Southampton Clinical Informatics Research Unit.

Only the Lead Surgeon at each site and authorized personnel should enter or change data in the electronic Case Report Forms (eCRFs) in ALEA. Further details regarding data collection and storage via the ALEA online platform are provided in Supplementary A6.

Quality of the data entered into the eCRF data fields will be controlled by limiting free text fields, drop-down options, and predefined data formats. Range checks for chosen fields will automatically appear where data points are outside of a prespecified range. Verification and explanation for unexplained data points will be required and will subsequently appear in a query log for the study team to check.

### Data validation

Data completeness from individual centers for all study fields should be 95% or greater. If data completeness is less than 95% then the local study team will be required to investigate. Failure to do so will be considered by the steering committee, and data from that center may not be included in the final analysis. Individual units will be asked to nominate an independent data validator as part of the study team who will review 20% of submitting patient files and 25% of data points within these submissions. The overall responsibility for data completeness and accuracy will rest with the Lead Consultant for that institution. It will also be possible to utilize Hospital Episodes Statistics data in order to identify all patients admitted to individual centers for ARS during the time period and measure case ascertainment to ensure completeness of data entry.

### Data analysis

Results will be prepared in accordance with the guidelines as set by the STROBE (Strengthening the Reporting of Observational Studies in Epidemiology) statement for observational studies.^31^ Data will be collated and analyzed in clinically relevant categories, and chi-square tests used, where appropriate, to detect differences in proportions between groups. Procedures performed for missing data, if identified, will include multiple imputations.

Results will be analyzed to compare adherence to the established audit standards as detailed in Table 1.

An initial pilot data collection period has been completed at five UK hospitals (Musgrove Park, North Bristol, University Hospitals Bristol, Southampton, and Brighton) in February 2020 in order to test the feasibility of the collection of proposed data points using the ALEA eCRF. Sites will be required to preregister for the audit and obtain local study approval as per institution policy prior to commencement of the study.

### Patient and Public Involvement

During the development of this study, the patient charities Heartburn Cancer UK (www.heartburncanceruk.org), Action Against Heartburn (https://www.actionagainstheartburn.org.uk/), and Barrett’s Wessex (www.barrettswessex.org.uk) have all offered support to this multicenter audit. Heartburn Cancer UK has reviewed the study protocol during the development stage and was able to provide specific guidance regarding how patient priorities, experience, and preferences may be incorporated into the study design. Having reviewed the proposed study design these charitable groups have agreed there would not be any additional burden of intervention or time upon patients. These charities will be involved in the ongoing plans for methods of dissemination of study results.

### Proposed Study Timeline

Due to the current challenges faced by the health services in the UK by COVID-19, the start of this study is being delayed until normal elective operating practice is resumed nationally with a planned start-date of 1 April 2021. The proposed study timeline is detailed below:

- April 2021 to April 2022—main study data collection period.
- July 2022—main study 90-day follow-up ends.
- September 2022—central data submission anticipated to be complete.
- December 2022—anticipated that independent data validation completed.
- April 2022—initial data analysis anticipated to be complete.

### Authorship

Manuscript preparation following data analysis will be undertaken by a writing committee. All members of the ARROW protocol writing group, steering committee, lead surgeons, and team members for individual centers will be Pubmed citable collaborators as part of the ARROW Study Group. Units who fail to submit data, or whose data are incomplete (as outlined above) will be excluded from the authorship list. Prior to publication, each lead surgeon will be responsible for collating a list of authors from their site.

### Ethics and Dissemination

This study will follow the previously reported approach for dissemination amongst surgical research collaboratives. Local teams will retain access to their own data to facilitate local quality improvement. The full dataset will be reported at national and international scientific congresses and will contribute to peer-reviewed publications and national quality improvement initiatives.

## DISCUSSION

Variation in surgical practice and outcomes after elective surgical intervention are an important quality metric within healthcare. This study will identify and explore variation in process and outcome after ARS within the UK using a collaborative cohort methodology. This will provide granular data regarding the practice of ARS at a national level. The results generated by this study will facilitate local and national quality improvement initiatives and generate new possibilities for future research in anti-reflux interventions.

## Supplementary Material

arrow_appendix_1_doaa129

arrow_appendix_2_doaa129

-arrow_appendix_3_doaa129

arrow_appendix_4_doaa129

arrow_appendix_5_REVISIONS_Changes_Tracked_doaa129

arrow_appendix_6_doaa129

arrow_appendix_5_REVISIONS_Clean_Version_doaa129

## Author Statement

This protocol has been authored as a collaborative by the ARROW study writing group members (RW, TW, NB, JMF, MW, ACC, SH, SRM, SR, ML, MH, SJ). All members contributed to study design, drafting of the manuscript, and final approval of the protocol for publication. Other members of the study group (SH, FN, AM, ROM, RK, RZ, SH, PI, TU) have all significantly contributed to the development of this study.

## Notes

Study group: Natalie S. Blencowe, Andrew Currie, John M. Findlay, Scott Harris, Marianne Hollyman, Steve Hornby, Phil Ireland, Shameen Jaunoo, Renol Koshy, Megan Lloyd, Anantha Mahadevan, Sheraz R. Markar, Fergus Noble, Robert O’Neill, Saqib Rahman, Tim Underwood,Robert Walker, Tom Wiggins, Michael Wilson, Roxanna Zakeri

## Notes

Design: Multicenter, prospective audit of current clinical practice.
