# Supplementary material for: A multicenter prospective audit to investigate the current management of patients undergoing anti-reflux surgery in the UK: Audit & Review of Anti-Reflux Operations & Workup
Source: Dis Esophagus. 2021 Jan 16;34(7):doaa129. doi: 10.1093/dote/doaa129 (PMC8522793; doi:10.1093/dote/doaa129)
Supplement: arrow_appendix_1_doaa129 [file arrow_appendix_1_doaa129.docx]

***APPENDIX 1***

Novel interventions for GORD and their mechanism of action

| **Novel Interventions for GORD** | | |
| --- | --- | --- |
| **LINX** | Magnetic Sphincter Augmentation | Ethicon, Somerville, NJ, USA |
| **Stretta** | Radio-frequency stimulation of Lower Oesophageal Sphincter | Restech, Houston, TX, USA |
| **EsophyX** | Transoral/Endoscopic Incisionless Fundoplication | EndoGastricSolutions, Redmond, WA, USA |
| **IM RefluxStop** | Implant to augment angle of His | Implantica, Baar, Switzerland |
