# Supplementary material for: A multicenter prospective audit to investigate the current management of patients undergoing anti-reflux surgery in the UK: Audit & Review of Anti-Reflux Operations & Workup
Source: Dis Esophagus. 2021 Jan 16;34(7):doaa129. doi: 10.1093/dote/doaa129 (PMC8522793; doi:10.1093/dote/doaa129)
Supplement: arrow_appendix_2_doaa129 [file arrow_appendix_2_doaa129.docx]

**APPENDIX 2**

Details of clinical recommendations considered for inclusion as audit standards and reasons for exclusion where applicable.

| **Audit Standard** | **Included as audit measure** | **Reason for exclusion** |
| --- | --- | --- |
| **ICARUS Guidelines^24^** | | |
| ARS can be considered for patients with typical symptoms of heartburn, with a good response to PPI. | No | Measures of patient selection for ARS not being collected as part of ARROW. |
| Patients with functional heartburn and patients with eosinophilic oesophagitis are poor candidates for ARS. | No | Measures of patient selection for ARS not being collected as part of ARROW. |
| Patients with morbid obesity and patients with substance abuse are not excluded from ARS. | No | Measures of patient selection for ARS not being collected as part of ARROW. |
| Endoscopy (during the last year) is mandatory prior to referral for ARS. | Yes | N/A |
| Patients with GORD symptoms and a hiatal hernia, Barrett’s oesophagus or erosive oesophagitis grade B or higher at endoscopy are good candidates for ARS. | No | Measures of patient selection for ARS not being collected as part of ARROW. |
| Patients without erosive oesophagitis are not excluded from ARS. | No | Measures of patient selection for ARS not being collected as part of ARROW. |
| There is no need to obtain routine biopsies of the distal oesophagus in patients considered for ARS. | No | Not possible to develop audit standard to measure adherence against. |
| A barium X-ray should be obtained in patients with a suspicion of a hiatal hernia or short oesophagus when considered for ARS. | No | Potential subjective interpretation of guidelines would make measurement of audit standard potentially inaccurate. |
| Patients with GORD symptoms and a hiatal hernia on X-ray are good candidates for ARS. | No | Measures of patient selection for ARS not being collected as part of ARROW. |
| Patients with GORD symptoms and a para-oesophageal hernia on X-ray are good candidates for ARS in addition to para-oesophageal hernia repair. | No | Measures of patient selection for ARS not being collected as part of ARROW. |
| A short oesophagus on barium X-ray does not preclude the patient from ARS. | No | Measures of patient selection for ARS not being collected as part of ARROW. |
| Oesophageal manometry and oesophageal pH monitoring (±impedance) are mandatory prior to referral for ARS (pH monitoring only mandatory in patients with non-erosive reflux diseas). | Yes | N/A |
| Patients with normal pH-monitoring off PPI are poor candidates for ARS. | No | Measures of patient selection for ARS not being collected as part of ARROW. |
| Response to baclofen does not enhance patient eligibility to ARS. | No | Measures of patient selection for ARS not being collected as part of ARROW. |
| There is no need to asses gastric emptying rate in patients considered for ARS. | No | Not possible to develop audit standard to measure adherence against. |
|  | | |
| **British Society of Gastroenterology (BSG) Guidelines^23^** | | |
| Any staff member performing manometry or reflux monitoring should either be fully trained or accredited by the AGIP in this procedure or supervised by a fully trained and accredited practitioner. | No | Oesophageal physiology quality measures beyond the scope of ARROW study. |
| All patients undergoing manometry for the investigation of dysphagia should undergo at least one form of adjunctive testing (eg: larger volumes of water, solid/viscous swallows or a test meal). | No | Oesophageal physiology quality measures beyond the scope of ARROW study. |
| All patients undergoing manometry to investigate dysphagia should have previously undergone endoscopy and mucosal biopsy. | No | Only patients with reflux symptoms (rather than dysphagia alone) included and performance of endoscopy prior to ARS captured separately. |
| All patients undergoing reflux monitoring should have manometry to guide probe placement | No | Oesophageal physiology quality measures beyond the scope of ARROW study. |
| All patients undergoing ARS should have manometry to exclude major oesophageal motility disorders. | Yes | N/A |
| All impedance recordings should be manually edited to ensure accurate reflux symptom association | No | Oesophageal physiology quality measures beyond the scope of ARROW study. |
| All patients should have at least two methods of symptom association assessed (eg: SAP and SI). | No | Oesophageal physiology quality measures beyond the scope of ARROW study. |
| All patients should undergo reflux monitoring prior to ARS. | No | Included for patients with non-erosive reflux disease as per ICARUS guidelines. |

|  | | |
| --- | --- | --- |
| **The Provision Of Services For Upper Gastrointestinal Surgery Association of Upper GI Surgeons (AUGIS)^22^** | | |
| Unit rate of conversion to open surgery during ARS of <5% | Yes | N/A |
| Unit readmission rate following ARS of <10% within 30 days of surgery | Yes | N/A |
| Unit re-operation rate at 30 days following ARS of <5% | Yes | N/A |
| Laparoscopic ARS minimum activity per surgeon of >5 per annum | No. | Important to gain accurate clinical representation of current United Kingdom practice in this audit which units will voluntarily participate in. Therefore necessary to also include any potential low-volume clinical centres for ARS. |
