# Supplementary material for: A multicenter prospective audit to investigate the current management of patients undergoing anti-reflux surgery in the UK: Audit & Review of Anti-Reflux Operations & Workup
Source: Dis Esophagus. 2021 Jan 16;34(7):doaa129. doi: 10.1093/dote/doaa129 (PMC8522793; doi:10.1093/dote/doaa129)
Supplement: -arrow_appendix_3_doaa129 [file arrow_appendix_3_doaa129.docx]

**APPENDIX 3**

**Surgeon Survey**

**Registration**

Name of surgeon:

Email address:

Please enter all hospitals the surgeon works for and select the main one:

- Hospital name …
- County …
- Main one:

**Surgeon Survey**

Date completed …

Please select job role:

- Consultant
- Associate Specialist

Year first appointed as a consultant/associate specialist:

Primary practice:

- Benign upper GI
- Bariatric
- OG resectional
- HPB resectional
- General Surgery
- Colorectal

Please estimate how many anti-reflux surgery cases you perform per year:

- NHS …
- Private …

Which preoperative investigations would you consider **compulsory** for anti-reflux surgery:

Tick all that apply

- OGD
- Upper GI contrast study
- CT
- 24-hour pH monitoring
- Wireless pH monitoring (BRAVO)
- 24-hour impedance monitoring
- High resolution manometry
- Standard resolution manometry
- Other

Where is your oesophageal physiology testing (including pH monitoring, manometry and impedance) performed?

- NHS laboratory situated within your Trust
- NHS laboratory at another hospital Trust
- External laboratory
- Not sure

Which of the following procedures might you undertake for GORD?

- Fundoplication:
- LINX:
- Stretta:
- EsophyX:
- Roux-en-Y gastric bypass
- RefluxStop™
- Other: (please name)

Which of the following procedures might you perform when doing a fundoplication?

- Nissen posterior 360^o^
- Toupet posterior 270^o^
- Toupet posterior 180^o^
- Dor anterior 180^o^
- Watson anterior 120^o^
- Partial anterior 90^o^
- Collis:
- Other

Do you tailor the type of wrap performed for each individual?

- Yes, based on manometry findings
- Yes, based on clinical symptoms
- Never

Which procedures might you perform as an intended day-case?

- Fundoplication:
- LINX:
- Stretta:
- EsophyX:
- Roux-en-Y gastric bypass
- None performed as intended day case procedure:
- Other: (please name)

What criteria do you apply for same-day discharge following anti-reflux surgery (other than standard requirements for day-case surgery)?

- Tolerance of solid oral intake
- Tolerance of liquid oral intake
- Proximity of patient residence from hospital
- Surgery completed by a specific time
- Other (please give details): ________________________________________

Do you divide the short-gastric vessels?

- Routinely
- Selectively
- Never

Do you perform an anterior cruroplasty?

- Routinely
- Selectively
- Never

Do you perform a posterior cruroplasty?

- Routinely
- Selectively
- Never

Do you perform a Collis oesophageal lengthening procedure?

- Routinely
- Selectively
- Never

Do you repair over a bougie or orogastric tube?

- Routinely
- Selectively
- Never

Do you maintain a prospective database of your anti-reflux surgery practice?

- Yes
- No

Do you record severity/symptom or quality of life scores pre-operatively?

- Routinely
- Selectively
- Never

Do you record severity/symptom or quality of life scores post-operatively?

- Routinely
- Selectively
- Never

Post-Op workup: Do you carry out a routine post-operative assessment of the wrap with:

OGD

- Yes
- No

Upper GI contrast study

- Yes
- No

Do you believe that anti-reflux surgery (other than gastric bypass for reflux disease) is effective for patients with obesity?

- Yes
- No

Do you believe that increasing patient BMI potentially influences the likelihood of a successful outcome with anti-reflux surgery (other than gastric bypass for reflux disease)?

- Yes
- No

What is the upper limit of a patient’s BMI that you would perform anti-reflux surgery (other than gastric bypass for reflux disease)?

- <25
- <30
- <32
- <35
- <40
- <45
- <50
- No limit
- Alternative criteria used

Do you request patients with obesity complete a pre-operative liver shrinkage diet prior to surgery?

- Yes
- No
- Selectively

If yes do you use specific clinical or BMI criteria for advising patients to complete this?

- Do not utilise
- BMI>32
- BMI>35
- BMI>40
- BMI>45
- BMI>50
- Central pattern obesity
- Clinical examination of abdominal wall stiffness in RUQ
- Alternative criteria used
- No specific criteria

Do you discharge patients home with an anti-emetic?

- Yes
- No
- Selectively

Do you discharge patients home with opioid analgesia?

- Yes
- No
- Selectively

Are you the lead surgeon for the Arrow Audit at any institution?

- Yes
- No

(if Yes opens Institutional survey)

**Institutional survey**

Estimated number of cases of anti-reflux surgery performed annually at your institution: …

Number of surgeons completing anti-reflux surgery at your institution: …

Is oesophago-gastric resectional surgery performed at your institution?

- Yes
- No

Is bariatric surgery performed at your institution?

- Yes
- No

Does your institution have set funding criteria for anti-reflux procedures? If so, please describe or supply a copy of funding criteria?

- Yes
- No
- Details: ________________________________________________________

Is your institution required to apply to the local Clinical Commissioning Group for funding of anti-reflux surgery on an individual patient basis?

- Yes
- No

Preop workup: Which of the following does your institution have access to for the investigation of reflux:

- OGD
- Upper GI contrast study
- CT
- 24-hour pH monitoring
- Wireless pH monitoring (eg. BRAVO™)
- 24-hour impedance monitoring
- High resolution oesophageal manometry
- Standard resolution oesophageal manometry

Where is your oesophageal physiology testing (including pH monitoring, manometry and impedance) performed?

- NHS laboratory situated within your Trust
- NHS laboratory at another hospital Trust
- External laboratory
- Not sure

Does your institution maintain a prospective database of anti-reflux surgery practice?

- Yes
- No

Does your institution have access to a benign UGI MDT to discuss patients prior to anti-reflux surgery?

- Routinely
- Selectively
- Only prior to revision surgery
- Never

If your institution holds a benign UGI MDT is this held:

- Locally
- Regionally (in person)
- Regionally (via tele-link)

If your institution holds a benign MDT who are your core members?

- UGI Surgeons
- Gastroenterologists
- Radiologist
- Physiologist
- UGI Nurse Specialist
- Other (details below)
- Details of other team members: _________________________________

Does your institution give a standardised pre-operative information sheet/booklet to patients?

- Yes
- No

Does your institution have a standardised advice sheet for post-operative diet?

- Yes
- No

Do all surgeons in your institution follow the same post-operative diet protocol?

- Yes
- No

What routine clinical follow up does your institution perform?

- In-person clinic appointment (doctor)
- In-person clinic appointment (nurse)
- Telephone clinic (doctor)
- Telephone clinic (nurse)
- None
