# Supplementary figures and images for: A multicenter prospective audit to investigate the current management of patients undergoing anti-reflux surgery in the UK: Audit & Review of Anti-Reflux Operations & Workup
Source: Dis Esophagus. 2021 Jan 16;34(7):doaa129. doi: 10.1093/dote/doaa129 (PMC8522793; doi:10.1093/dote/doaa129)

**APPENDIX 4**


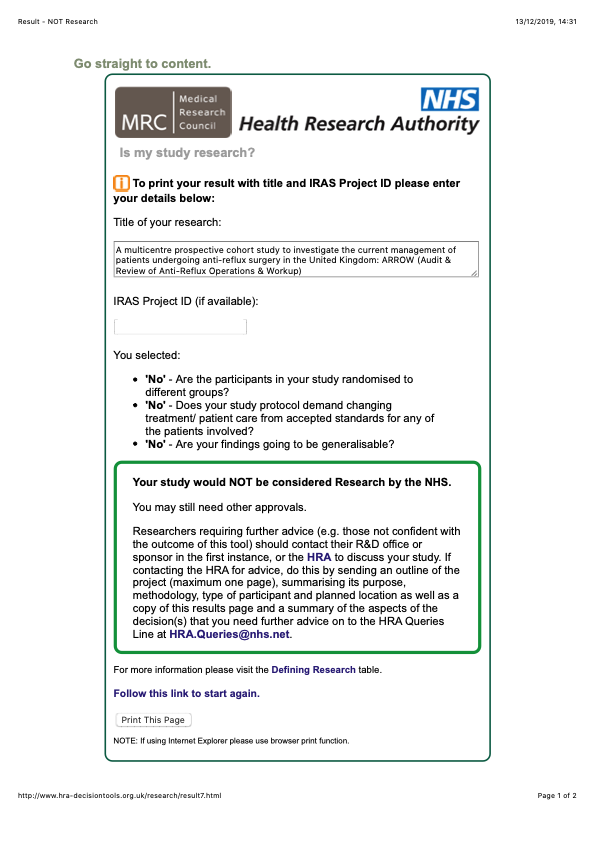

Supplement: arrow_appendix_4_doaa129 [file arrow_appendix_4_doaa129.docx]
