# Supplementary material for: A multicenter prospective audit to investigate the current management of patients undergoing anti-reflux surgery in the UK: Audit & Review of Anti-Reflux Operations & Workup
Source: Dis Esophagus. 2021 Jan 16;34(7):doaa129. doi: 10.1093/dote/doaa129 (PMC8522793; doi:10.1093/dote/doaa129)
Supplement: arrow_appendix_5_REVISIONS_Changes_Tracked_doaa129 [file arrow_appendix_5_revisions_changes_tracked_doaa129.docx]

A**PPENDIX 5**

**Case Report Form (CRF)**

[Completed via online portal through ALEA]

**1. Registration**

Date of Registration: _ _/_ _/_ _ _ _

Patient Age: _______

**2. Patient History**

2.1 Demographics

Sex: Male

Female

ASA: I

II

III

IV

V

Height: _____ metres

Weight: ____ Kg

OR

BMI (derived): ______

Previous Thoracic Surgery: Yes

No

Unknown

If Yes Thoracoscopic

Open

Both

Previous abdominal surgery: Yes

No

Unknown

If yes: Laparoscopic

Open

Both

Unknown

Any Co-morbidities: Yes

No

Unknown

If yes:

Charlson comorbidity index:

Acute myocardial infarction

Cancer

Cerebral vascular accident

Congestive heart failure

Connective tissue disorder

Dementia

Diabetes

Diabetes complications

HIV

Liver disease

Metastatic cancer

Paraplegia

Peptic ulcer

Peripheral vascular disease

Pulmonary disease

Renal disease

Severe liver disease

Total score: ____

Smoking status:

Current smoker

Ex-smoker

Never smoked

Unknown

Vaping status:

Current vaper

Ex- vaper

Never vaped

Unknown

2.2 Referrals

Date of referral:

Source of referral: GP/Primary care

Gastroenterology

ENT

Respiratory

Transplant

Other hospital specialist

Unknown

If not primary care:

Was this referral from:

The same hospital

Another hospital

Duration of symptoms at referral:

0 - 6 months

6 - 12 months

1 - 2 years

2 - 5 years

> 5 years

> 10 years

Current use of PPI: Yes (continuous)

Yes (intermittent)

No

Unknown

Current use of H_2_ Antagonist (eg Ranitidine):

Yes (continuous)

Yes (intermittent)

No

Unknown

2.3 Symptoms

Heartburn

Daytime heartburn: Yes

No

Unknown

Nocturnal heartburn: Yes

No

Unknown

Positional heartburn: Yes

No

Unknown

Documented as responsive to PPI:

Yes

No

Unknown

Regurgitation

Daytime regurgitation: Yes

No

Unknown

Nocturnal regurgitation: Yes

No

Unknown

Positional regurgitation: Yes

No

Unknown

Other Symptoms

Epigastric/chest pain: Yes

No

Unknown

Sleep disturbance: Yes

No

Unknown

Dysphagia: Yes

No

Unknown

Chronic cough: Yes

No

Unknown

Chronic laryngitis: Yes

No

Unknown

Dental erosions: Yes

No

Unknown

Any Patient Reported Outcome Measures used:

No

GERDQ

GSFQ

Other – please specify

2.4 Workup

OGD performed: Yes

No

Unknown

Date of most recent OGD: …

OGD findings:

Barrett’s Oesophagus: Yes

No

Unknown

Prague Classification Circumferential length …… cm

Maximum extent ….. cm

Oesophagitis: No

Yes - Grade A

Yes - Grade B

Yes - Grade C

Yes - Grade D

Yes - Grade Unknown

Unknown

Stricture: Yes

No

Unknown

Hiatus Hernia: Yes

No

Hiatus Hernia Size: Small<2cm

Medium 2-5cm

Large >5cm

>50% intrathoracic Stomach

Total intrathoracic Stomach

24h pH monitoring performed: Yes

No

Incomplete

Total time pH <4 ….hrs ….mins or ….%

Symptom Correlation Index .…% or value 0-1

Symptom Association Probability .…% or value 0-1

DeMeester Score ….

Impedance monitoring performed Yes

No

Incomplete

Impedance results:

Acid reflux

Weak-acid reflux

Non-acidic reflux

Manometry performed: Yes

No

Incomplete

If yes: High resolution

Standard resolution

Peristalsis:

Normal

Weak

Absent

Lower Oesophageal Sphincter Pressure: …

Integrated Relaxation Pressure IRP: …

Contrast swallow performed: Yes

No

CT performed: Yes (oral contrast)

Yes (no contrast)

No

Hiatus Hernia Size: Small<2cm

Medium 2-5cm

Large >5cm

>50% intrathoracic Stomach

Total intrathoracic Stomach

Other intra-abdominal organs within hiatus hernia

None

Colon

Pancreas

Spleen

Other

Other investigation performed: [Details] __________________

**3. Surgery**

3.1 – Revisional surgery

Is this a revisional procedure?

Yes

No

If Yes:

How long ago was the primary procedure?

< 6 months

6-12 months

1-5 years

>5 years

>10 years

What was the primary procedure?

Fundoplication

LINX

EsophyX  Stretta

Gastric bypass

Other – please specify

Has this patient had previous revisions?

Yes

No

Indication for this revision:

Recurrence of symptoms

Dysphagia

3.2 Primary Indication for Surgery

What is the primary indication for surgery? (Tick one only)

Symptoms not sufficiently controlled by PPI

Not compliant with PPI

Side effects with PPI

Symptoms adequately controlled but patient prefers surgical management:

Intra-oesophageal complications of GORD

Lung transplant patient

Non-oesophageal symptoms/complications of GORD

Info: Chronic cough, dental erosions, aspiration pneumonia etc.

Other: [Details] __________________

3.3 Surgery

Date of operation: ­_ _/_ _/_ _ _ _

Grade of primary surgeon: ST3-6

ST7-8

Staff Grade

Associate Specialist

Post CCT Fellow

Consultant

Was the procedure a training case: Yes(in part)

Yes(in full)

No

Unknown

Was the operation as part of an unplanned admission:

Yes

No

Unknown

Procedure performed: Fundoplication

LINX

EsophyX

Stretta

Gastric bypass

Other – please specify

Was the planned procedure performed: Yes

No

Unknown

If No: Procedure planned: Fundoplication

LINX

EsoPhyx

Stretta

Gastric bypass

Other – please specify

Was the procedure planned as a day case: Yes

No

Unknown

Procedure Specific Details

Procedure Duration: <1hr

1-2hrs

2-3hrs

3-4hrs

>4hrs

Fundoplication intra-operative details [Additional drop-down menu]

Approach: Open

Laparoscopic

Robotic

Endoscopic

Lap converted to open

Robotic converted to lap

Robotic converted to open

Endo converted to lap

Endo converted to open

Posterior cruroplasty: Yes

No

Anterior cruroplasty: Yes

No

Suture material: Absorbable

Non-absorbable

Sizing of repair: Bougie/OG tube

Visual

Mesh used: No

Biologic

Synthetic – non-absorbable

Synthetic – absorbable

Composite – synthetic

Composite – synthetic + biological

If mesh used:

Mesh fixation: Absorbable sutures

Non-absorbable sutures

Metal tacks

Non-metal tacks – absorbable

Non-metal tacks – non-absorbable

Hepatic vagal fibres preserved: Yes

No

Anterior vagus preserved: Yes

No

Posterior vagus preserved: Yes

No

Division of short gastrics: Yes

No

Wrap anterior/posterior: Anterior

Posterior

Wrap 180/270/360: 90

120

180

270

360

Oesophageal stitch: Yes

No

Diaphragmatic stitch: Yes

No

Collis (oesophageal lengthening procedure): Yes

No

Other modifications: [Details] …

Roux-en-Y Gastric Bypass:

Alimentary limb length …

Biliary limb length …

LINX intra-operative details [Additional drop-down menu]

LINX Size: 10

11

12

13

14

15

16

17

18

All Procedures:

Intraoperative complications: Yes

No

Pneumothorax (requiring drain): Yes

No

Major visceral injury: No

Oesophagus

Stomach

Spleen

Liver

Colon

Small Bowel

Heart/ Pericardium

Other …

Major vascular injury: No

Aorta

Inferior Vena Cava

Other …

Estimated blood loss:

Minimal

100-500ml

500-1000ml

1-2l

>2l

3.3 Post-Operative

Date of discharge: …

Delayed discharge? Yes

No

If yes: Reason: Dysphagia

Inadequate oral intake

Vomiting

Pain

Pneumonia

Pneumothorax

Urinary retention

Social

Other – please specify

Investigations: None

Laparoscopy

Contrast swallow

CT

OGD

CXR

Other – please specify

(able to select multiple options)

Interventions: None

Antibiotics

Chest drain

Return to theatre

If ticked – please specify ….

- Date of operation …

Pneumatic dilatation

Other – please specify

(able to select multiple options)

Complications? Yes

No

If yes: Number of Complications (as per Clavien Dindo classification^1^):

(on index admission) Grade 1 …

Grade 2 …

Grade 3a …

Grade 3b …

Grade 4a …

Grade 4b …

Grade 5 …

Readmission within 90 days? (Including admission for day case procedure/investigation)

No
Yes

If yes:

Date of readmission …

Date of discharge from readmission …

Reason: Dysphagia

Inadequate oral intake

Vomiting

Pain

Pneumonia

Pneumothorax

Urinary retention

Social

Other – please specify

Investigations: None

Laparoscopy

Contrast swallow

CT

OGD

CXR

Other – please specify

(able to select multiple options)

Interventions: None

Antibiotics

Chest drain

Return to theatre

If ticked – please specify ….

- Date of operation …

Pneumatic dilatation

Other – please specify

(able to select multiple options)

Complications? Yes

No

If yes:

Number of Complications (as per Clavien Dindo classification^1^):

(on index admission) Grade 1 …

Grade 2 …

Grade 3a …

Grade 3b …

Grade 4a …

Grade 4b …

Grade 5 …

**4. Outcomes**

Follow-up within 90 days of operation:

Routine Outpatient-Doctor

Routine Outpatient-Nurse

Unplanned re-presentation

Telephone - Doctor

Telephone - Nurse

No follow-up planned

Did not attend follow-up

If followed up:

Date of first follow-up: _ _/_ _/_ _ _ _

Discharged after first follow-up: Yes

No

Any Patient Reported Outcome Measures used:

No

GERDQ

GSFQ

Other – please specify

Resolution of symptoms? Yes

No

Partial

Ongoing PPI used: Yes

No

**References**

1. Dindo D, Demartines N, Clavien PA. Classification of surgical complications: a new proposal with evaluation in a cohort of 6336 patients and results of a survey. Ann Surg. 2004;240(2):205-13.
