# Supplementary material for: A multicenter prospective audit to investigate the current management of patients undergoing anti-reflux surgery in the UK: Audit & Review of Anti-Reflux Operations & Workup
Source: Dis Esophagus. 2021 Jan 16;34(7):doaa129. doi: 10.1093/dote/doaa129 (PMC8522793; doi:10.1093/dote/doaa129)
Supplement: arrow_appendix_6_doaa129 [file arrow_appendix_6_doaa129.docx]

**APPENDIX 6**

**Data storage technical details**

ALEA eCRF is an electronic Case Report Forms service for the data collection in clinical trials. It provides a comprehensive, user friendly forms service which can be used with a standard browser running on any computer connected to the internet. The system has been validated and has been certified by registered auditors to be in compliance with regulation, such as the FDA’s CFR 21 Part 11.

ALEA consists of a study design (SD) component and a data management (DM) component. During setup and maintenance of the study, the SD component is used to create or modify the design of the study. The DM component exists on a test/development, acceptance and production instance. The test/development instance provides an environment to test the setup and modifications for the CIRU (Clinical Informatics Research Unit) programmers while the acceptance instance is used by the client for user acceptance testing. The production instance is used once the study is live. These environments are physically isolated, and do not share data and accounts.

SD and the test/development environment of data management are hosted in Amsterdam. The acceptance and production environments of DM are hosted in Den Bosch. This location is a secured, ISO 27001 certified data centre operated by InterConnect BV in Den Bosch, the Netherlands. FormsVisions’ Quality Assurance includes formal disaster management procedures for management of issues related to the operational environment. Measurements include failover, local data recovery, and site recovery. Each physical server is equipped with RAID5 disk redundancy, redundant power supply and redundant network connectivity. The server facilities in Den Bosch include both hot standby and cold standby servers. Hot standby servers (DBSHV3 and DBSSQL2) allow for near-instant failover to a running server in case of physical server failure. In case of logical server failure, cold standby servers (DBSHV4, DBSHV5) provide local data recovery in case the site is operational. In case of site failure, the disaster recovery procedure provides transfer of all operational services to our hosting facilities in Amsterdam.
